# Bilirubin Concentration in Follicular Fluid Is Increased in Infertile Females, Correlates with Decreased Antioxidant Levels and Increased Nitric Oxide Metabolites, and Negatively Affects Outcome Measures of In Vitro Fertilization

**DOI:** 10.3390/ijms241310707

**Published:** 2023-06-27

**Authors:** Renata Mangione, Romina Pallisco, Gabriele Bilotta, Francesca Marroni, Valentina Di Pietro, Elena Capoccia, Giuseppe Lazzarino, Barbara Tavazzi, Giacomo Lazzarino, Pasquale Bilotta, Angela Maria Amorini

**Affiliations:** 1 Department of Basic Biotechnological Sciences, Intensive and Perioperative Clinics, Catholic University of Rome, Largo F. Vito 1, 00168 Rome, Italy; renata.mangione@unicatt.it; 2 Laboratory of Andrology and Embriology, Alma Res Fertility Center, Via Parenzo 12, 00198 Rome, Italy; laboratorio@almares.it (R.P.); bilotta.oblomov@gmail.com (G.B.); marronifrancesca@gmail.com (F.M.); elena.capoccia753@gmail.com (E.C.); 3 Neurotrauma and Ophthalmology Research Group, Institute of Inflammation and Aging, University of Birmingham, Birmingham B15 2TT, UK; 4 NIHR Surgical Reconstruction and Microbiology Research Centre, University Hospitals Birmingham NHS Foundation Trust, Birmingham B15 2TT, UK; 5 Division of Medical Biochemistry, Department of Biomedical and Biotechnological Sciences, University of Catania, Via S. Sofia 97, 95123 Catania, Italy; amorini@unict.it; 6 LTA—Biotech srl, Viale Don Orione 3D, 95047 Paternò, Italy; 7 Departmental Faculty of Medicine and Surgery, UniCamillus—Saint Camillus International University of Health and Medical Sciences, Via di Sant’Alessandro 8, 00131 Rome, Italy; barbara.tavazzi@unicamillus.org; 8 Service of Obstetrics and Gynecology, Alma Res Fertility Center, Via Parenzo 12, 00198 Rome, Italy; pasquale.bilotta@almares.it

**Keywords:** antioxidants, assisted reproduction techniques, bilirubin, fertility rates, follicular fluid, heme oxygenase, inflammation, oxidative/nitrosative stress, reactive oxygen species

## Abstract

In a previous study, we showed that various low-molecular-weight compounds in follicular fluid (FF) samples of control fertile females (CFF) have different concentrations compared to those found in FF of infertile females (IF), before and after their categorization into different subgroups, according to their clinical diagnosis of infertility. Using the same FF samples of this previous study, we here analyzed the FF concentrations of free and bound bilirubin and compared the results obtained in CFF, IF and the different subgroups of IF (endometriosis, EM, polycystic ovary syndrome, PCOS, age-related reduced ovarian reserve, AR-ROR, reduced ovarian reserve, ROR, genetic infertility, GI and unexplained infertility, UI). The results clearly indicated that CFF had lower values of free, bound and total bilirubin compared to the respective values measured in pooled IF. These differences were observed even when IF were categorized into EM, PCOS, AR-ROR, ROR, GI and UI, with EM and PCOS showing the highest values of free, bound and total bilirubin among the six subgroups. Using previous results of ascorbic acid, GSH and nitrite + nitrate measured in the same FF samples of the same FF donors, we found that total bilirubin in FF increased as a function of decreased values of ascorbic acid and GSH, and increased concentrations of nitrite + nitrate. The values of total bilirubin negatively correlated with the clinical parameters of fertilization procedures (number of retrieved oocytes, mature oocytes, fertilized oocytes, blastocysts, high-quality blastocysts) and with clinical pregnancies and birth rates. Bilirubin concentrations in FF were not linked to those found in serum samples of FF donors, thereby strongly suggesting that its over production was due to higher activity of heme oxygenase-1 (HO-1), the key enzyme responsible for bilirubin formation, in granulosa cells, or cumulus cells or oocytes of IF and ultimately leading to bilirubin accumulation in FF. Since increased activity of HO-1 is one of the main enzymatic intracellular mechanisms of defense towards external insults (oxidative/nitrosative stress, inflammation), and since we found correlations among bilirubin and oxidative/nitrosative stress in these FF samples, it may reasonably be supposed that bilirubin increase in FF of IF is the result of protracted exposures to the aforementioned insults evidently playing relevant roles in female infertility.

## 1. Introduction

The influence of the follicular fluid (FF) composition on both natural and in vitro fertilization has been clearly evidenced by tens of studies that have appeared in the literature in the last decades [1,2]. In particular, the composition of FF, in terms of low-molecular-weight compounds connected to various biochemical functions, is fundamental to allow correct oocyte maturation and development following either natural or in vitro fertilization [3,4,5].

Alterations of compounds related to energy metabolism, such as glucose and lactate, have been found in FF of infertile females suffering from different pathological conditions of infertility [6,7]. Also, decreased levels of FF antioxidants (ascorbate, reduced glutathione, coenzyme Q_10_) may be causative of infertility and are often associated with clear signatures of increased oxidative/nitrosative stress [8,9].

These biochemical modifications of FF composition are certainly the sum of different contributions from both the oocyte and the cumulus and granulosa cells. Under fertility conditions, the correct functioning of the different cell types involved in FF formation allows either the transport from the bloodstream to FF of nutrients and other relevant compounds (antioxidants, amino acids) or the countertransport of end products of oocyte catabolism (for instance, lactate and ammonia). Therefore, besides the crucial role in releasing correct hormonal signals, this complex cell system, oocyte–cumulus cell-granulosa cell, is definitely relevant to ensure the correct biochemical composition of FF, which is crucial to allow pregnancy.

Bilirubin is the yellow pigment originating as the end product of heme catabolism from the combined actions of heme oxygenase (HO) (the first, rate-limiting step forming biliverdin from heme) and of biliverdin reductase (the second step forming bilirubin from biliverdin) [10]. Although the majority of bilirubin formation occurs through the metabolism of the heme of hemoglobin (Hb) in the reticuloendothelial system [11], a significant proportion of bilirubin generation occurs ubiquitously in all cells of all tissues, from the metabolism of non-Hb heme released during the turnover of intracellular hemoproteins (for instance, cytochromes, catalase, peroxidases, nitric oxide synthases) [12].

In the liver, in order to increase its solubility and therefore favor its subsequent excretion, circulating bilirubin is conjugated with one or two molecules of glucuronic acid and again released into the bloodstream, where different bilirubin forms can be found: (i) unconjugated bilirubin (UB); (ii) unconjugated, albumin-bound bilirubin (UB-A); two isomers of bilirubin monoglucuronate (depending on which of the two –COOH of bilirubin the glucuronate is linked to) (BMG); bilirubin diglucuronate (in which both –COOH of the molecule are conjugated with glucuronic acid) (BDG). Accumulation of part of UB in the bile and deglucuronidation and transformation of BMG and BDG into products of excretion (stercobilin and urobilin) contribute to maintain under control the circulating levels of UB. In fact, whilst low UB levels are considered of great advantage for the organism in light of the antioxidant properties of bilirubin [13], excess of UB is deleterious for cell wellness [14,15].

As previously mentioned, intracellular heme catabolism is mainly dependent on the activity of HOx, the enzymes catalyzing the rate-limiting reaction of heme degradation. HO exists in two isoforms: heme oxygenase 1 (HO-1) and heme oxygenase 2 (HO-2). Whilst HO-2 is constitutively expressed in all human tissues (although in different amounts), HO-1 is the inducible isoenzyme that can be found in a lower number of tissues [16,17]. The expression of HO-1 is modulated by that of the nuclear factor erythroid-2-related factor 2 (Nrf2), activating the cell defense systems (including HO-1) against a variety of stressors [18,19,20]. Pathological conditions causing oxidative/nitrosative stress, inflammation and mitochondrial malfunctioning are capable of activating the Nrf2/HO-1 axis as a cell/tissue protective response to increase the probability of cell/tissue to survive the stressor. Hence, in cells/tissues undergoing a physical–chemical insult capable of triggering the aforementioned molecular alterations, the increase in expression and activity of HO-1 lead to augmented bilirubin formation in light of its antioxidant and signaling properties [21,22]. Therefore, levels of cell/tissue bilirubin may indicate the presence of stressing stimuli connected to pathological states and altered clinical conditions. In infertile females, oxidative/nitrosative stress and inflammation are associated with endometriosis (EM), polycystic ovary syndrome (PCOS) and also reduced ovarian reserve (ROR) [23,24,25,26,27,28], thus creating the pathological conditions for an excess formation of bilirubin in their FF.

Recently, we have shown that FF of infertile females (IF) affected by EM, PCOS, aged ROR (A-ROR), ROR, unexplained infertility (UI) and genetic infertility (GI), compared to control fertile females (CFF), had numerous alterations in terms of low-molecular-weight compounds representative of antioxidant defenses, nitrosative stress, energy metabolism and amino acid metabolism [29]. Using the same FF samples of this previous study, we here examined whether bilirubin concentration had different values in CFF and in IF, subsequently categorized into EM, PCOS, ROR, A-ROR, UI and GI. Results of the FF bilirubin levels were correlated with the water-soluble antioxidants and nitric oxide metabolites (nitrite + nitrate), previously measured in the same FF samples from the same FF donors [29], as well as with outcome measures of clinical pregnancy, in order to find potential correlates of clinical relevance.

## 2. Results

### 2.1. Concentration of Bilirubin in FF of Control Fertile and Pooled Infertile Females

**Figure 1:**
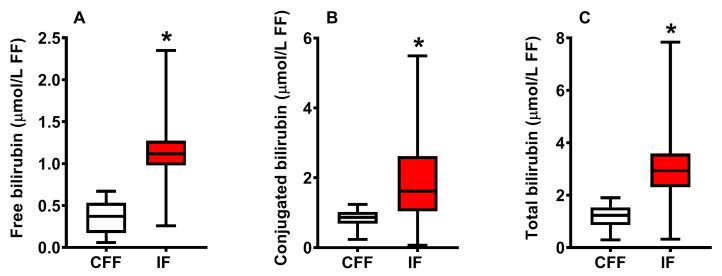
Box plots reporting median, 1st and 3rd quartiles, minimum and maximum values of the concentrations of free bilirubin (**A**), conjugated bilirubin (**B**) and total bilirubin (**C**) detected by HPLC in FF of 35 control fertile females (CFF) and 145 infertile females (IF) grouped in a single cohort of infertility. * Significantly different from CFF, *p* < 0.001.

The results illustrated in Figure 1 show the concentration of free (**A**), conjugated (**B**) and total (**C**) bilirubin in the group of control fertile females (CFF) and in that of infertile females (IF) grouped into a single cohort, independently of the clinical diagnosis of infertility.

Mean values of free, conjugated and total bilirubin in CFF were, respectively, 0.36 ± 0.18, 0.84 ± 0.24 and 1.20 ± 0.42 μmol/L FF, whilst values measured in FF of pooled CFF were, respectively, 1.15 ± 0.26 (+319%), 1.95 ± 1.01 (+232%) and 3.07 ± 1.20 (+256%) μmol/L FF. To evaluate whether the FF levels of the three forms of bilirubin might discriminate between CFF and pooled IF, we calculated the corresponding receiver operating characteristic (ROC) curves.

**Figure 2:**
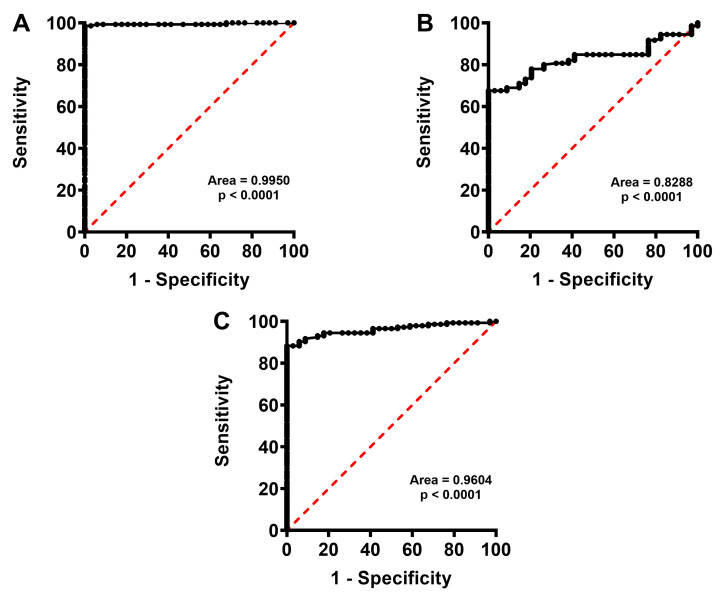
Receiver Operating Characteristic (ROC) curves calculated using the levels of free bilirubin (**A**), conjugated bilirubin (**B**) and total bilirubin (**C**) determined in FF samples of CFF and pooled IF.

As shown in Figure 2, calculations of ROC curves indicate that concentrations of each one of the three bilirubin forms in FF clearly clustered, with high sensitivity and specificity, CFF and pooled IF into two distinct groups. 

### 2.2. Concentration of Bilirubin in FF of CFF and IF Categorized According to Their Clinical Diagnosis of Infertility

**Figure 3:**
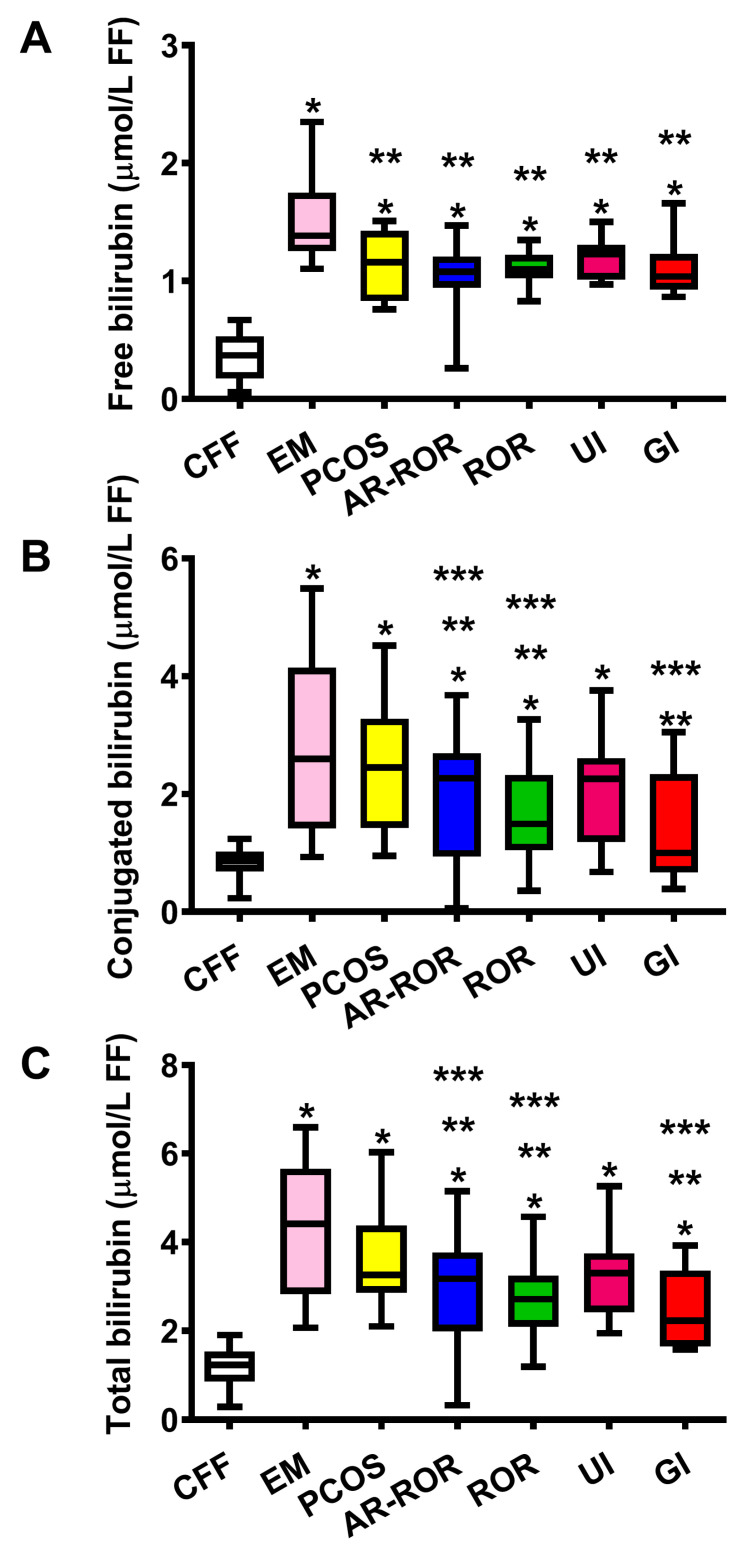
Box plots reporting median, 1st and 3rd quartiles, minimum and maximum values of the concentrations of free bilirubin (**A**), conjugated bilirubin (**B**) and total bilirubin (**C**) detected by HPLC in FF of 35 control fertile females (CFF) and 145 infertile females (IF), categorized into six subgroups according to the clinical diagnosis of infertility. EM = endometriosis (n = 19); PCOS = polycystic ovary syndrome (n = 14); A-ROR = aged-related reduced ovarian reserve (n = 58); ROR = reduced ovarian reserve (n = 29); UI = unidentified infertility (n = 14); GI = genetic infertility (n = 11). * Significantly different from CFF, *p* < 0.001; ** Significantly different from EM, *p* < 0.01; *** Significantly different from PCOS, *p* < 0.01.

To determine whether bilirubin levels were influenced by the differential clinical diagnoses of infertility, concentrations of the three bilirubin forms (unconjugated, conjugated and total) detected in FF of pooled IF were divided into six subgroups of EM, PCOS, A-ROR, ROR, UI and GI, depending on the results of the clinical assessment and the consequent assignment of each patient to one of the aforementioned subgroups. The results are illustrated in Figure 3.

The results illustrated in Figure 3 clearly indicate that each of the six subgroups, in which IF were divided, had values of free, conjugated and total bilirubin significantly higher than the corresponding values measured in CFF. Additionally, the data also show that IF patients belonging to the two subgroups of EM and PCOS had values of the three bilirubin forms higher than those determined in the remaining four IF subgroups.

### 2.3. Increased Concentration of Bilirubin in FF Correlates with Decreased Ascorbic Acid and GSH, and Increased Nitrite + Nitrate

**Figure 4:**
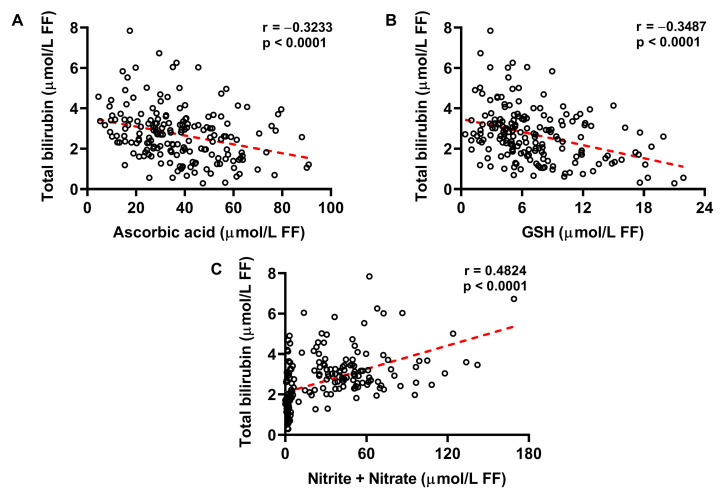
FF concentrations of total bilirubin as a function of FF ascorbic acid (**A**), GSH (**B**) and nitrite + nitrate (**C**), independently of the classification of FF donors into CFF or IF. Spearman’s correlation coefficients were significant in any of the aforementioned associations.

Taking advantage of the measurements of ascorbic acid, GSH, nitrite + nitrate (as stable end products of nitric oxide metabolism) previously carried out in the same FF samples from the same FF donors of both groups of CFF and IF [29], we evaluated the potential correlations among bilirubin FF concentrations and those of each of the aforementioned compounds in FF. As shown in Figure 4, increases in bilirubin FF values are associated with decreases in ascorbic acid (**A**) and GSH FF concentrations (**B**). Conversely, FF bilirubin levels increase as a function of increasing concentrations of nitrite + nitrate in FF. Altogether, these findings reinforce the concept that stressing conditions, such as those occurring under oxidative/nitrosative stress, are very probably responsible for bilirubin overproduction released in FF by accessory cells (cumulus cells, granulosa cells) and, possibly, by oocyte itself.

### 2.4. Concentration of Bilirubin in FF Correlates with Biological and Clinical Parameters of IVF and Successful Pregnancy

**Figure 5:**
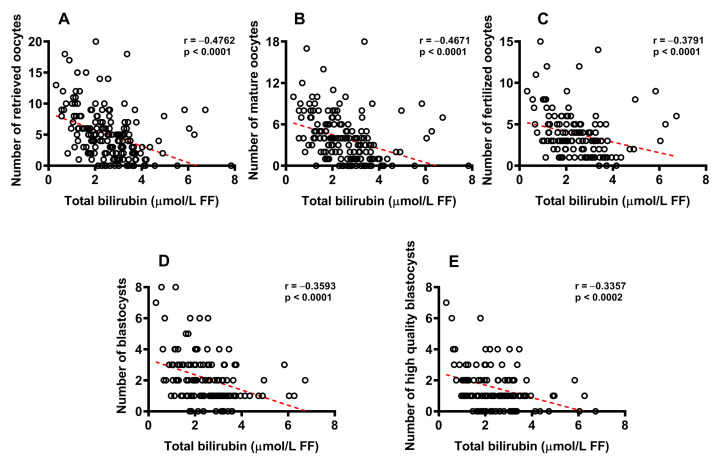
Linear correlations between the FF concentration of total bilirubin and number of retrieved oocytes (**A**), number of mature oocytes (**B**), number of fertilized oocytes (**C**), number of blastocysts (**D**) and number of high-quality blastocysts (**E**) in pooled FF donors, independently of their classification into CFF or IF. Spearman’s correlation coefficients were significant in any of the aforementioned associations.

As shown in Figure 5, in order to evaluate the potential influence on IVF efficiency, we plotted the numbers of retrieved oocytes (**A**), mature oocytes (**B**), fertilized oocytes (**C**), blastocysts (**D**) and high-quality blastocysts (E) as a function of the total bilirubin concentration determined in FF of donors, pooled into a single group independently of the assignment to the group of CFF or IF.

Highly significant values of the Spearman’s correlation coefficients were found when correlating concentration of total bilirubin in FF to the number of retrieved oocytes (r = −0.476; *p* < 0.0001), number of mature oocytes (r = −0.467; < 0.0001), number of fertilized oocytes (r = −0.379; *p* < 0.0001), number of blastocysts (r = −0.359; *p* < 0.0001) and number of high-quality blastocysts (r = −0.336; *p* < 0.0002).

In order to evaluate the effect on the number of pregnancies and live births, FF donors who underwent embryo transfer, independently of their classification into CFF or IF, were divided into two groups of pregnancy (n = 42) and no pregnancy (n = 68), and two groups of births (n = 27) and no births (n = 12). 

**Figure 6:**
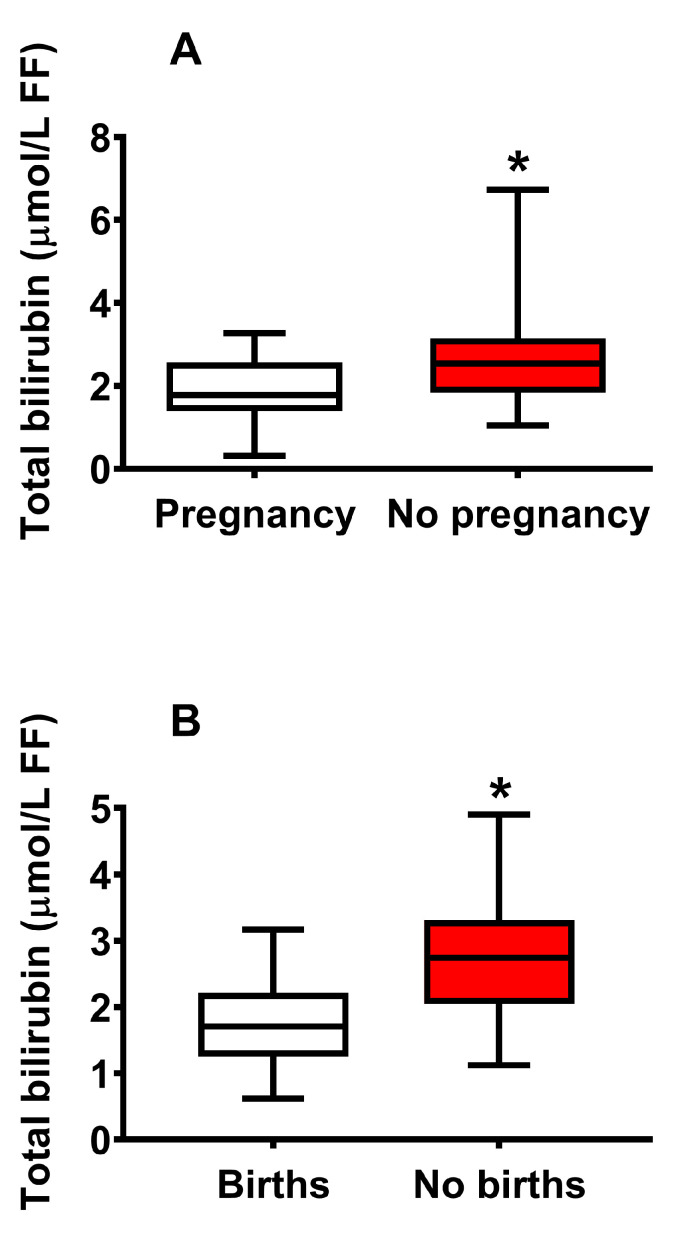
Box plots reporting median, 1st and 3rd quartiles, minimum and maximum values of the FF total bilirubin concentration in those FF donors who underwent embryo transfer and who had a clinical pregnancy (**A**) and who delivered healthy offspring (**B**). FF donors were pooled into a single group, independently of their classification into CFF or IF. * Significantly different from pregnancy and births, *p* < 0.001.

As shown in Figure 6, FF concentration of total bilirubin was higher either in FF donors with no pregnancy (**A**), or in FF donors with no live births (**B**).

## 3. Discussion

In a recent study, we observed that FF of IF had numerous alterations in various hydrophilic and hydrophobic low-molecular-weight compounds, compared to the values measured in CFF [29]. Furthermore, when IF were categorized according to the clinical diagnosis of infertility, different patterns of biochemical alterations were found in their FF samples [29]. Due to the variable-intensity yellowish color of FF and to previously published results [30,31], we reanalyzed FF samples of the aforementioned groups of 35 CFF and 145 IF [29], with the aim to determine the concentrations of free bilirubin, conjugated bilirubin and total bilirubin and their possible correlations with infertility. The results reported in the present study clearly evidenced that concentrations of FF bilirubin, either free, or conjugated or total, are associated with infertility, FF antioxidants, FF oxidative/nitrosative stress, unsuccessful IVF, clinical pregnancy and live births.

When considering the differences between CFF and pooled IF, we found that FF of infertile females had bilirubin concentrations (free, conjugated and total) 2–3 times higher than those measured in FF of fertile females. Since the concentrations of the majority of the low-molecular-weight compounds detectable in FF reflect, under physiological conditions, those found in serum [32], we hypothesized that IF may have high levels of circulating bilirubin, thus explaining the higher concentrations in their FF, compared to those in FF of CFF. The retrospective analysis allowed the recovery of a limited number of FF donors who performed routine blood analysis (in the days before pickup) that also included the determination of serum bilirubin. In this subset of FF donors, free, conjugated and total bilirubin in CFF serum (n =17) were, respectively, 4.49 ± 3.81, 5.62 ± 2.63 and 10.11 ± 4.44 μmol/L, whilst levels measured in IF serum (n = 59) were, respectively, 3.47 ± 2.29, 5.97 ± 2.77 and 9.44 ± 3.06 μmol/L, thereby indicating no differences in the circulating concentrations in any of the three bilirubin forms between fertile and infertile females.

With this in mind, it is highly conceivable to hypothesize that at least part of the bilirubin in FF is not derived through a simple mechanism of equilibration between the circulating (higher than FF) and the FF (lower than serum) levels, but rather that it may plausibly be the result of the intracellular production by the oocyte and accessory cells (cumulus and granulosa cells), releasing their production of bilirubin in FF. To this regard, the presence and relevance of both the inducible (HO-1) and the constitutive (HO-2) forms of heme oxygenase, the enzyme responsible for the first reaction of the heme catabolic pathway, have clearly been demonstrated in female reproductive cells [33,34,35].

Although the levels of expression of HO-1 are associated with defense cell mechanisms towards ROS-mediated damages [36,37], Bergandi et al. [38] and Canosa et al. [39] showed that higher expressions of HO-1 are associated with poorer embryo quality and development. Indeed, the increased expression of a defense mechanism, such as the increased HO-1 expression, is the result of the cell response towards stressing conditions. That is, high levels of HO-1 indicate that cells have been exposed to physical–chemical insults for a sufficient period of time to induce the activation of defense mechanisms, including HO-1 overexpression. It is certainly conceivable to suspect that, under conditions of increased HO-1 expression, the formation of the end product of the combined activity of HO-1 and biliverdin reductase, i.e., bilirubin, is increased.

The results reported in the present study, showing higher bilirubin concentrations in FF of infertile females, particularly in FF of those donors diagnosed for EM and PCOS, strongly suggest that this biochemical evidence is due to insurgence of pathological conditions, potentially causing increased ROS production and consequent oxidative/nitrosative stress, and ultimately inducing a cell response (of cumulus and granulosa cells) aimed to increase antioxidant defenses [40,41]. The strong correlations between bilirubin and ascorbic acid, bilirubin and GSH, bilirubin and nitrite + nitrate (with bilirubin increasing in FF as a function of decreasing ascorbic acid and GSH, and increasing nitrite + nitrate) demonstrate the link between bilirubin FF concentrations and oxidative/nitrosative stress and corroborates the hypothesis that this phenomenon is the result of a defense mechanism of the female reproductive cell system. It is well known that oxidative/nitrosative stress triggers overexpression of the Nrf2-HO-1 axis, which is of paramount importance to ensure adequate cell protection from oxidative insults. Hence, the increases in gene and protein HO-1 expressions are generally considered as indicators of stressing conditions triggering the endogenous mechanisms aimed to increase probability of cell survival under pathological conditions [42,43,44].

In this light, it is worth recalling that the same FF samples from infertile donors used in this study had also decreased concentrations of α-tocopherol and coenzyme Q_10_, and increased values of malondialdehyde and 8-hydroxy-deoxyguanosine compared to the values measured in FF of controls [29], strongly corroborating the indication that the increased FF bilirubin levels in infertile females are the result of tentative protective mechanisms against pathological conditions increasing inflammation [45] and oxidative/nitrosative stress [46].

In conclusion, although the present data do not allow for determining the cell source implicated in the bilirubin increase in FF of infertile females, they show for the first time that this phenomenon is connected with oxidative/nitrosative stress and with all the biological measures determining IVF success, particularly clinical pregnancies and live births. Since a bilirubin increase in FF is associated with the increase of parameters reflecting oxidative/nitrosative stress and the decrease in water-and fat-soluble antioxidants, it is conceivable to hypothesize that the administration of selected antioxidants to infertile females may be beneficial to increase success of IVF. Studies aimed to determine the efficacy of these treatments (through the biochemical analysis of FF before and after antioxidant therapies) and to determine HO-1 expression/activity in oocyte accessory cells are in progress.

## 4. Materials and Methods 

### 4.1. Patients’ Characteristics and Protocols for Ovarian Stimulation

The study was conducted according to the Declaration of Helsinki for Medical Research involving Human Subjects. At the time of enrollment, all FF donors signed informed written consents to participate in this study.

As previously indicated [29], FF donors were recruited at the Alma Res Fertility Centre (Rome, Italy) from September 2018 to January 2020, with the approval of the Alma Res Ethical Committee (approval number AREC0818FF). Infertile females (IF, n = 145) did not achieve pregnancy after at least one year of unprotected sexual intercourse with the same partner. Fertile females of couples with fertility problems because of a male factor alone, who agreed to donate their FF for the analysis, represented the control groups (CFF, n = 35). The inclusion in this group was performed after a scrupulous clinical evaluation excluding any female involvement and only when laboratory evidence allowed a clear diagnosis of male infertility. Exclusion from the study occurred in the case of a mechanical barrier in the female reproductive system, previous history of cancer and premature ovarian failure. Categorization of IF was carried out through gynecological assessment, including hormone analysis, hysterosalpingography, and transvaginal ultrasound to evaluate uterine cavity and antral follicle counts. After assessment, IF could be divided into the subgroups of endometriosis (EM, n = 19), polycystic ovary syndrome (PCOS, n = 14), age-related reduced ovarian reserve (AR-ROR, n = 58), reduced ovarian reserve (ROR, n = 29), unexplained infertility (UI, n = 14) and genetic infertility (GI, n = 11). Details for the assignment of IF to the different subgroups, with particular attention to those of EM, PCOS and GI, have previously been described [29].

In CFF, and in the subgroups of PCOS, UI and GI, ovarian stimulation was induced by the administration of GnRH antagonist and menotropin started on day 2 after the beginning of the cycle, followed by GnRH antagonist and recombinant human chorionic gonadotropin (r-HCG) at the end of the stimulation. In the subgroups of EM, AR-ROR and ROR, the ultrashort protocol for ovarian stimulation was applied (ultrashort flare GnRH analogue starting on day 1 after the beginning of the cycle, followed by menotropin administration on day 2). In vitro fertilization (IVF) or intracytoplasmic sperm injection (ICSI) was performed in CFF and IF, on the basis of male factor involvement in infertility and the type of infertility. Conventional IVF was carried out when donors were diagnosed with unexplained infertility or endometriosis, whilst ICSI was performed when previous IVF failure or suboptimal fertilization occurred.

To minimize external variables affecting analytical results, all FF donors were assessed in order to determine their dietary pattern and lifestyle (all nonsmokers, no one with alcohol or drug dependence, mild-to-moderate physical activity). Questionnaires to determine the adherence to the Mediterranean diet [47] and to evaluate the levels of physical activity [48] were administered to each FF donor before clinical assessment. Those patients who had a supplementation of adjuvants/nutraceuticals during the 3 months before the enrollment were excluded from the study.

### 4.2. Follicle Recovery, FF Preparation, Embryo Culture and Assessment of Successful Fertilization

Transvaginal, ultrasound-assisted follicle aspiration (approximate size of ≥18 mm) was carried out 36 h after the injection of r-HCG. FF samples were prepared as previously described [29]. Briefly, pooled FF from the same donor were centrifuged (10 min at 1500× *g* at room temperature) and 500 μL of the supernatants were then processed for the analysis of fat-soluble metabolites. Blood contamination caused the discarding of FF samples.

The preparation of oocytes for insemination and conditions for their culture are those applied in our previous study [29]. The number and the quality (the latter determined using the Gardner blastocyst grading system) of developed blastocysts were assessed 5 days after the insemination, followed by transfer or vitrification. The number of retrieved oocytes, mature oocytes, fertilized oocytes, blastocysts and high-quality blastocysts (grading ≥ 4AA) were determined. The number of clinical pregnancies and of healthy offspring were taken into account for those FF donors who decided on embryo transfer.

### 4.3. Samples Processing and HPLC Assay of Bilirubin in the Fat-Soluble Fraction of FF Samples

FF supernatants (500 μL) were light-protected, deproteinized by the addition of 1 mL of HPLC-grade acetonitrile, vortexed for 60 s and placed in a water bath for 1 h at 37 °C. As previously shown, this procedure allows the quantitative recovery of fat-soluble compounds in biological samples [49,50,51,52]. Protein-free FF were obtained by centrifugation at 20,690× *g* for 15 min at 4 °C and then saved at −80 °C until the HPLC analysis of fat-soluble compounds, including free and bound bilirubin.

The analysis of FF for the quantification of bilirubin was performed using a Surveyor HPLC apparatus (Thermo Fisher Scientific, Rodano, Milan, Italy). A highly sensitive 5 cm light-path flow cell diode array, set up for acquisition between 200 and 550 nm wavelengths, was used for bilirubin detection. Aliquots of protein-free FF acetonitrile extracts (100 μL) were loaded onto a Hypersil Gold C-18, 150 × 4.6 mm, 3 μm particle size column (Thermo Fisher Scientific, Rodano, Milan, Italy), provided with its own guard columns. Quantification of free and conjugated bilirubin in FF extracts was performed by comparing retention times and area of the peaks with those of chromatographic runs containing known concentrations of ultrapure standards of free and bound bilirubin, using the ChromQuest^®^ software package (5.0 version) provided by the HPLC manufacturer.

### 4.4. Statistical Analysis

Statistical analysis was performed by using the GraphPad Prism program, release 8.0.1. The normality of distribution was tested by the Kolmogorov–Smirnov test. For all continuous variables, minimum, 25% percentiles, medians, 75% percentiles, maximum, range, 95% CI of medians, means, standard deviations, lower and upper 95% CI of means were calculated. Differences among the two groups of CFF and pooled IF were displayed by using the Mann–Whitney U test, and those among CFF and the 6 subgroups of IF divided according to the clinical diagnosis (EM, PCOS, AR-ROR, ROR, UI, GI) were determined by the 1-way ANOVA, followed by the Tukey test, as the post hoc test. A *p* value of less than 0.05 was considered statistically significant. The power calculations of the study’s sample size indicated a minimum of 33 FF sample/group to have a 90% power with an α < 0.05. To evaluate the effects size, the Hedge’s g values of each comparison between variables under consideration were calculated and are reported in the Supplementary Materials Tables S1–S14. Correlations among the concentrations of bilirubin and ascorbic acid, GSH, nitrite + nitrate in FF, as well as those among bilirubin in FF andclinical parameters of fertilization procedures (number of retrieved oocytes, mature oocytes, fertilized oocytes, blastocysts, high-quality blastocysts) were calculated using the Spearman’s correlation coefficient.

## Data Availability

Data available on request due to privacy and ethical restrictions.

## Supplementary Materials

The following supporting information can be downloaded at: https://www.mdpi.com/article/10.3390/ijms241310707/s1.
