# Supplementary material for: Bilirubin Concentration in Follicular Fluid Is Increased in Infertile Females, Correlates with Decreased Antioxidant Levels and Increased Nitric Oxide Metabolites, and Negatively Affects Outcome Measures of In Vitro Fertilization
Source: Int J Mol Sci. 2023 Jun 27;24(13):10707. doi: 10.3390/ijms241310707 (PMC10342083; doi:10.3390/ijms241310707)
Supplement: Supplementary file 1 [file ijms-24-10707-s001.zip › ijms-2440184-supplementary.pdf]

**Table S1.** Descriptive statistics of the free bilirubin levels determined in FF of CFF and in pooled IF.

| | CFF | IF |
|---|---|---|
| **Number of values** | 35 | 145 |
| **Minimum** | 0.05820 | 0.2600 |
| **25% Percentile** | 0.1725 | 0.9785 |
| **Median** | 0.3700 | 1.118 |
| **75% Percentile** | 0.5325 | 1.273 |
| **Maximum** | 0.6700 | 2.350 |
| **Range** | 0.6118 | 2.090 |
| **95% CI of median** | | |
| **Actual confidence level** | 97.57% | 95.41% |
| **Lower confidence limit** | 0.2000 | 1.079 |
| **Upper confidence limit** | 0.4400 | 1.180 |
| **Mean** | 0.3591 | 1.150 |
| **Std. Deviation** | 0.1831 | 0.2557 |
| **Std. Error of Mean** | 0.03140 | 0.02124 |
| **Lower 95% CI of mean** | 0.2952 | 1.108 |
| **Upper 95% CI of mean** | 0.4229 | 1.192 |

**Table S2.** Descriptive statistics of the conjugated bilirubin levels determined in FF of CFF and in pooled IF.

| | CFF | IF |
|---|---|---|
| **Number of values** | 35 | 145 |
| **Minimum** | 0.2300 | 0.06000 |
| **25% Percentile** | 1.240 | 5.490 |
| **Median** | 1.010 | 5.430 |
| **75% Percentile** | | |
| **Maximum** | | |
| **Range** | 97.57% | 95.41% |
| | 0.7400 | 1.507 |
| **95% CI of median** | 0.9900 | 2.330 |
| **Actual confidence level** | | |
| **Lower confidence limit** | 0.8350 | 1.953 |
| **Upper confidence limit** | 0.2442 | 1.095 |
| | 0.04189 | 0.09094 |
| **Mean** | | |
| **Std. Deviation** | 0.7498 | 1.773 |
| **Std. Error of Mean** | 0.9202 | 2.132 |
| | 0.2300 | 0.06000 |
| **Lower 95% CI of mean** | 1.240 | 5.490 |
| **Upper 95% CI of mean** | 1.010 | 5.430 |

**Table S3.** Descriptive statistics of the total bilirubin levels determined in FF of CFF and in pooled IF.

| | CFF | IF |
|---|---|---|

| | | |
|---|---|---|
| **Number of values** | 35 | 145 |
| **Minimum** | 0.2900 | 0.3200 |
| **25% Percentile** | 1.900 | 7.840 |
| **Median** | 1.610 | 7.520 |
| **75% Percentile** | | |
| **Maximum** | | |
| **Range** | 97.57% | 95.41% |
| | 0.9800 | 2.740 |
| **95% CI of median** | 1.460 | 3.131 |
| **Actual confidence level** | | |
| **Lower confidence limit** | 1.196 | 3.071 |
| **Upper confidence limit** | 0.4219 | 1.197 |
| | 0.07235 | 0.09942 |
| **Mean** | | |
| **Std. Deviation** | 1.049 | 2.874 |
| **Std. Error of Mean** | 1.344 | 3.267 |
| | 0.2900 | 0.3200 |
| **Lower 95% CI of mean** | 1.900 | 7.840 |
| **Upper 95% CI of mean** | 1.610 | 7.520 |

**Table S4.** Effect size of free, conjugated and total bilirubin levels detected in FF of CFF and in pooled IF.

| | **Hedges' g values of Free bilirubin** | **Hedges' g values of Conjugated bilirubin** | **Hedges' g values of Total bilirubin** |
|---|---|---|---|
| **CFF versus IF** | 3.245* | 1.125** | 1.717*** |

*Huge effect size

**Large effect size

***Very large effect size

**Table S5.** Descriptive statistics of the free bilirubin levels determined in FF of CFF and in IF categorized into the corresponding categories of EM, PCOS, AR-ROR, ROR, UI and GI according to the clinical diagnosis of infertility.

| | CFF | EM | PCOS | AR-ROR | ROR | UI | GI |
|---|---|---|---|---|---|---|---|
| **Number of values** | 35 | 19 | 14 | 58 | 29 | 14 | 11 |
| **Minimum** | 0.05820 | 1.105 | 0.7600 | 0.2600 | 0.8300 | 0.9700 | 0.8650 |
| **25% Percentile** | 0.1725 | 1.252 | 0.8300 | 0.9418 | 1.023 | 1.013 | 0.9270 |
| **Median** | 0.3700 | 1.384 | 1.160 | 1.079 | 1.100 | 1.230 | 1.039 |
| **75% Percentile** | 0.5325 | 1.750 | 1.425 | 1.210 | 1.222 | 1.310 | 1.230 |
| **Maximum** | 0.6700 | 2.350 | 1.510 | 1.470 | 1.348 | 1.500 | 1.660 |
| **Range** | 0.6118 | 1.245 | 0.7500 | 1.210 | 0.5180 | 0.5300 | 0.7950 |
| **95% CI of median** | | | | | | | |
| **Actual confidence level** | 97.57% | 98.08% | 97.75% | 95.60% | 97.06% | 97.87% | 98.83% |
| **Lower confidence limit** | 0.2000 | 1.252 | 0.8200 | 1.008 | 1.033 | 1.000 | 0.9070 |
| **Upper confidence limit** | 0.4400 | 1.750 | 1.440 | 1.110 | 1.202 | 1.310 | 1.270 |
| **Mean** | 0.3591 | 1.478 | 1.115 | 1.065 | 1.110 | 1.200 | 1.110 |
| **Std. Deviation** | 0.1831 | 0.3385 | 0.2857 | 0.2034 | 0.1401 | 0.1722 | 0.2249 |
| **Std. Error of Mean** | 0.03140 | 0.07765 | 0.07925 | 0.02718 | 0.02517 | 0.04306 | 0.06781 |
| **Lower 95% CI of mean** | 0.2952 | 1.315 | 0.9419 | 1.010 | 1.058 | 1.108 | 0.9589 |
| **Upper 95% CI of mean** | 0.4229 | 1.641 | 1.287 | 1.119 | 1.161 | 1.292 | 1.261 |

**Table S6.** Descriptive statistics of the conjugated bilirubin levels determined in FF of CFF and in IF categorized into the corresponding categories of EM, PCOS, AR-ROR, ROR, UI and GI according to the clinical diagnosis of infertility.

| | CFF | EM | PCOS | AR-ROR | ROR | UI | GI |
|---|---|---|---|---|---|---|---|
| **Number of values** | 35 | 19 | 14 | 58 | 29 | 14 | 11 |
| **Minimum** | 0.2300 | 0.9320 | 0.9500 | 0.06000 | 0.3600 | 0.6800 | 0.3890 |
| **25% Percentile** | 0.6850 | 1.416 | 1.425 | 0.9393 | 1.043 | 1.188 | 0.6690 |
| **Median** | 0.8650 | 2.595 | 2.450 | 2.267 | 1.494 | 2.260 | 1.000 |
| **75% Percentile** | 1.020 | 4.150 | 3.280 | 2.694 | 2.320 | 2.610 | 2.340 |
| **Maximum** | 1.240 | 5.490 | 4.520 | 3.680 | 3.264 | 3.760 | 3.050 |
| **Range** | 1.010 | 4.558 | 3.570 | 3.620 | 2.904 | 3.080 | 2.661 |
| **95% CI of median** | | | | | | | |
| **Actual confidence level** | 97.57% | 98.08% | 97.75% | 95.60% | 97.06% | 97.87% | 98.83% |
| **Lower confidence limit** | 0.7400 | 1.416 | 1.410 | 1.400 | 1.063 | 1.050 | 0.6390 |
| **Upper confidence limit** | 0.9900 | 4.150 | 3.550 | 2.386 | 1.647 | 2.630 | 2.800 |
| **Mean** | 0.8350 | 2.644 | 2.510 | 1.853 | 1.608 | 2.084 | 1.408 |
| **Std. Deviation** | 0.2442 | 1.465 | 1.175 | 1.030 | 0.7229 | 0.9673 | 0.9293 |
| **Std. Error of Mean** | 0.04189 | 0.3362 | 0.3258 | 0.1376 | 0.1298 | 0.2418 | 0.2802 |
| **Lower 95% CI of mean** | 0.7498 | 1.937 | 1.800 | 1.577 | 1.343 | 1.568 | 0.7832 |
| **Upper 95% CI of mean** | 0.9202 | 3.350 | 3.220 | 2.129 | 1.873 | 2.599 | 2.032 |

**Table S7.** Descriptive statistics of the total bilirubin levels determined in FF of CFF and in IF categorized into the corresponding categories of EM, PCOS, AR-ROR, ROR, UI and GI according to the clinical diagnosis of infertility.

| | CFF | EM | PCOS | AR-ROR | ROR | UI | GI |
|---|---|---|---|---|---|---|---|
| **Number of values** | 35 | 19 | 14 | 58 | 29 | 14 | 11 |
| **Minimum** | 0.2900 | 2.070 | 2.100 | 0.3230 | 1.190 | 1.940 | 1.580 |
| **25% Percentile** | 0.8600 | 2.831 | 2.855 | 1.988 | 2.087 | 2.418 | 1.640 |
| **Median** | 1.230 | 4.407 | 3.260 | 3.174 | 2.716 | 3.300 | 2.230 |
| **75% Percentile** | 1.530 | 5.656 | 4.370 | 3.772 | 3.245 | 3.750 | 3.360 |
| **Maximum** | 1.900 | 6.595 | 6.030 | 5.145 | 4.570 | 5.260 | 3.920 |
| **Range** | 1.610 | 4.525 | 3.930 | 4.822 | 3.380 | 3.320 | 2.340 |
| **95% CI of median** | | | | | | | |

| | | | | | | | |
|---|---|---|---|---|---|---|---|
| **Actual confidence level** | 97.57% | 98.08% | 97.75% | 95.60% | 97.06% | 97.87% | 98.83% |
| **Lower confidence limit** | 0.9800 | 2.831 | 2.820 | 2.540 | 2.126 | 2.250 | 1.620 |
| **Upper confidence limit** | 1.450 | 5.656 | 4.730 | 3.340 | 2.995 | 3.770 | 3.920 |
| **Mean** | 1.194 | 4.121 | 3.625 | 2.918 | 2.718 | 3.286 | 2.518 |
| **Std. Deviation** | 0.4204 | 1.532 | 1.265 | 1.136 | 0.7465 | 1.014 | 0.8854 |
| **Std. Error of Mean** | 0.07209 | 0.3514 | 0.3508 | 0.1518 | 0.1341 | 0.2534 | 0.2670 |
| **Lower 95% CI of mean** | 1.047 | 3.383 | 2.860 | 2.614 | 2.444 | 2.746 | 1.923 |
| **Upper 95% CI of mean** | 1.341 | 4.859 | 4.389 | 3.222 | 2.991 | 3.826 | 3.113 |

**Table S8.** Effect size of the comparison of free, conjugated and total bilirubin levels detected in FF of CFF and in IF categorized into the corresponding categories of EM, PCOS, AR-ROR, ROR, UI and GI according to the clinical diagnosis of infertility.

| | Hedges' g values of Free bilirubin | Hedges' g values of Conjugated bilirubin | Hedges' g values of Total bilirubin |
|---|---|---|---|
| **CFF versus EM** | 4.504* | 2.042* | 3.045* |
| **CFF versus PCOS** | 3.544* | 2.556* | 3.221* |
| **CFF versus AR-ROR** | 3.668* | 1.219** | 1.844* |
| **CFF versus ROR** | 4.552* | 1.494** | 2.583* |
| **CFF versus UI** | 4.685* | 2.275* | 3.281* |
| **CFF versus GI** | 3.911* | 1.161** | 2.365* |

*Huge effect size

**Very large effect size

**Table S9.** Descriptive statistics of the total bilirubin levels determined in FF donors with and without clinical pregnancy.

| | Pregnancy | No pregnancy |
|---|---|---|
| **Number of values** | 42 | 68 |
| **Minimum** | 0.3200 | 1.050 |
| **25% Percentile** | 1.388 | 1.835 |
| **Median** | 1.770 | 2.535 |
| **75% Percentile** | 2.564 | 3.153 |
| **Maximum** | 3.270 | 6.730 |
| **Range** | 2.950 | 5.680 |
| **95% CI of median** | | |
| **Actual confidence level** | 95.64% | 96.15% |
| **Lower confidence limit** | 1.500 | 2.100 |
| **Upper confidence limit** | 2.230 | 2.920 |
| **Mean** | 1.907 | 2.634 |
| **Std. Deviation** | 0.7428 | 1.151 |

| | | |
|---|---|---|
| **Std. Error of Mean** | 0.1146 | 0.1396 |
| **Lower 95% CI of mean** | 1.676 | 2.355 |
| **Upper 95% CI of mean** | 2.139 | 2.913 |

**Table S10.** Effect size of total bilirubin levels detected in FF donors with and without clinical pregnancy.

| | **Hedges' g value of total bilirubin** |
|---|---|
| **Pregnancy versus No pregnancy** | 0.716* |

*Medium-to-large effect size

**Table S11.** Descriptive statistics of the total bilirubin levels determined in FF donors with and without clinical pregnancy.

| | **Pregnancy** | **No pregnancy** |
|---|---|---|
| **Number of values** | 27 | 12 |
| **Minimum** | 0.6200 | 1.120 |
| **25% Percentile** | 1.250 | 2.048 |
| **Median** | 1.710 | 2.745 |
| **75% Percentile** | 2.220 | 3.314 |
| **Maximum** | 3.170 | 4.900 |
| **Range** | 2.550 | 3.780 |
| **95% CI of median** | | |
| **Actual confidence level** | 98.08% | 96.14% |
| **Lower confidence limit** | 1.260 | 1.960 |
| **Upper confidence limit** | 2.070 | 3.350 |
| **Mean** | 1.720 | 2.768 |
| **Std. Deviation** | 0.6314 | 0.9838 |
| **Std. Error of Mean** | 0.1215 | 0.2840 |
| **Lower 95% CI of mean** | 1.470 | 2.143 |
| **Upper 95% CI of mean** | 1.970 | 3.393 |

**Table S12.** Effect size of total bilirubin levels detected in FF donors with and without clinical pregnancy.

| | **Hedges' g value of total bilirubin** |
|---|---|
| **Pregnancy versus No pregnancy** | 1.392* |

*Very large-to-huge effect size

**Table S13.** Results of the evaluation of the diet patterns of the two groups of FF donors (CFF and IF), using the Mediterranean Diet Scale proposed by Trichopoulou et al. (Trichopoulou, A.; Costacou, T.; Bamia, C.; Trichopoulos, D.Adherence to a Mediterranean diet and survival in a Greek population.*N Engl J Med***2003**,*348,*2599-2608. doi: 10.1056/NEJMoa025039). A questionnaire was administered to all participants at the time of enrollment, adopting the criteria of evaluation suggested in the aforementioned study.

| | **MDS 0-3 Number of subjects (%)** | **MDS 4-5 Number of subjects (%)** | **MDS 6-9 Number of subjects (%)** |
|---|---|---|---|
| **CFF (n = 35)** | 3 (8.6%) | 14 (40.0%) | 18 (51.4%) |
| **IF (n = 145)** | 10 (6.9%) | 65 (44.8%) | 70 (48.3%) |

MDS = Mediterranean Diet Score

MDS 0-3 = low adherence to Mediterranean diet

MDS 4-5 = moderate adherence to Mediterranean diet

MDS 0-3 = high adherence to Mediterranean diet

**Table S14.** Results of the evaluation of the physical activity (energy expenditure) of the two groups of FF donors (CFF and IF), using the attribution of Metabolic Equivalents of Task (MET)to different common physical activities described by Jetté et al. (Jetté M, Sidney K, Blümchen G. Metabolic equivalents (METS) in exercise testing, exercise prescription, and evaluation of functional capacity.*Clin Cardiol***1990,***13,*555-565. doi: 10.1002/clc.4960130809.).

| | **MET ≤ 3 Number of subjects (%)** | **MET 3-6 Number of subjects (%)** | **MET > 6 Number of subjects (%)** |
|---|---|---|---|
| **CFF (n = 35)** | 5 (14.3%) | 22 (62.9%) | 8 (22.9%) |
| **IF (n = 145)** | 26 (17.9%) | 100 (69.0%) | 19 (13.1%) |

MET = Metabolic Equivalents of Task

MET ≤ 3 = Sedentary-to-light active subjects

MET 3-6 = Moderately active subjects

MET > 6 = Highly active subjects
